# Peptide aptamers as new tools to modulate clathrin-mediated internalisation — inhibition of MT1-MMP internalisation

**DOI:** 10.1186/1471-2121-11-58

**Published:** 2010-07-23

**Authors:** Rochana D Wickramasinghe, Paul Ko Ferrigno, Christian Roghi

**Affiliations:** 1 MRC Cancer Cell Unit, Hutchison/MRC Research Centre, Hills Road, University of Cambridge, Cambridge, CB2 0XZ, UK; 2 University of Cambridge, Department of Oncology, Cambridge Research Centre, Li Ka Shing Centre, Robinson Way, Cambridge, CB2 0RE, UK; 3 Leeds Institute of Molecular Medicine, St James' University Hospital, University of Leeds, Leeds LS7 9TF, UK

## Abstract

**Background:**

Peptide aptamers are combinatorial protein reagents that bind to targets with a high specificity and a strong affinity thus providing a molecular tool kit for modulating the function of their targets *in vivo*.

**Results:**

Here we report the isolation of a peptide aptamer named swiggle that interacts with the very short (21 amino acid long) intracellular domain of membrane type 1-metalloproteinase (MT1-MMP), a key cell surface protease involved in numerous and crucial physiological and pathological cellular events. Expression of swiggle in mammalian cells was found to increase the cell surface expression of MT1-MMP by impairing its internalisation. Swiggle interacts with the LLY^573 ^internalisation motif of MT1-MMP intracellular domain, thus disrupting the interaction with the μ2 subunit of the AP-2 internalisation complex required for endocytosis of the protease. Interestingly, swiggle-mediated inhibition of MT1-MMP clathrin-mediated internalisation was also found to promote MT1-MMP-mediated cell migration.

**Conclusions:**

Taken together, our results provide further evidence that peptide aptamers can be used to dissect molecular events mediated by individual protein domains, in contrast to the pleiotropic effects of RNA interference techniques.

## Background

Peptide aptamers (PAs) are small, artificially engineered proteins conceptually similar to antibodies [1]. PAs consist of a stable, ideally inert scaffold protein with an inserted constrained peptide moiety. This in effect presents a small peptide surface within the tertiary structure of the scaffold which serves as the binding site for a target protein. In contrast to most of the more than 40 non-antibody scaffolds described to date [2], PAs are usually isolated by yeast-two hybrid screening of large libraries of PAs that contain random peptide inserts against a bait protein of interest. Selection of PAs in eukaryotic cells *in vivo *may allow the identification of interactors that are more easily transferable to mammalian cells than interactors identified using *in vitro *techniques such as phage-display. PA technology is well established, with PAs showing biological activity against a wide variety of proteins from different organisms, including the human and *D. melanogaster *Cdk2 proteins [1,3], the *E. coli *thymidylate synthase (ThyA) protein [4], the E6 and E7 proteins from human papilloma virus (HPV) [5,6], the human EGF receptor [7], and the transcription factors Stat3 [8] and the BCL-6 [9]. Importantly, some PAs have also been found to block functions of their target proteins *in vivo*, such as human Cdk2 [10], *D. melanogaster *Cdk1 and 2 [3], E2F [11], p53 [12], Stat3 [8], Nr-13 [13], and BCL-6 [9].

Membrane-type 1 Matrix Metalloproteinase (MT1-MMP, also known as MMP-14), is a member of the large MMP family of enzymes. MT1-MMP plays a major role in the dynamic remodelling of the extra-cellular matrix (ECM) and has been reported to directly degrade a broad spectrum of ECM proteins, including collagen types I, II, and III, fibronectin, laminin 1, laminin 5, fibrin, and aggrecan [14,15]. MT1-MMP has also been reported to activate proMMP-2 and proMMP-13 [16,17], thereby indirectly increasing its proteolytic repertoire on or near the cell surface. The protease also plays a role in the processing of a growing number of membrane proteins, including, for example, CD44 [18], transglutaminase [19], the integrin αV chain [20] or syndecan 1 [21] thus modulating cell signalling and the cellular functions mediated by these molecules.

MT1-MMP has been implicated in a wide spectrum of physiological and pathological cellular functions [22,23]. MT1-MMP expression, well documented in many tumours, has been correlated with key *in vitro *and *in vivo *processes of tumour progression including angiogenesis [24], cell migration and invasion [25], cell growth [26] and metastatic spread [27,28]. Inhibition or silencing of the protease has been found to significantly reduce the invasive phenotype of tumour cells implicating a leading role for MT1-MMP in such processes [25,29].

MT1-MMP is a type I transmembrane protein with a very short intracellular domain (ICD) of just 21 amino acids. The MT1-MMP ICD has been reported to be required for cell migration and invasion [30-33] as well as tumour growth [34]. The identification of proteins interacting with the MT1-MMP ICD, such as MTCBP-1 [35], and glCqR [36] have also helped in defining new localisations and cellular functions for this protease. The MT1-MMP ICD has also been implicated in the internalisation [31] and the recycling of the protease to the cell surface [37]. Consistent with this, MT1-MMP ICD has been reported to interact with the μ2 subunit of the AP-2 complex [31] as well as with caveolin-1 [38]

To date, crucial information on the cellular function of the intracellular domain of the protease has been obtained following exogenous expression of mutant MT1-MMP ICD constructs [31,39,38,37,41,33] or constructs with a partially or completely deleted ICD [30,42,26,43,40,34]. In order to assess the role of the MT1-MMP ICD without using exogenously truncated or mutated forms of the protease, we decided to make use of PA technology.

In this study, we identify and characterize a PA, named swiggle, which interacts with the 21 amino acid ICD of MT1-MMP. Expression of swiggle in human cells was found to stimulate MT1-MMP mediated cell migration. Detailed analysis of the phenotypic effect of swiggle revealed that the PA inhibits internalization of MT1-MMP resulting in the accumulation of the protease at the cell surface. Our data indicate that swiggle interacts with the LLY^573 ^motif in the MT1-MMP ICD and competes with the μ2 subunit of the AP-2 complex in cells, thereby inhibiting the endocytosis of MT1-MMP.

## Results

### Isolation of peptide aptamers that interact with the MT1-MMP ICD

**Figure 1:**
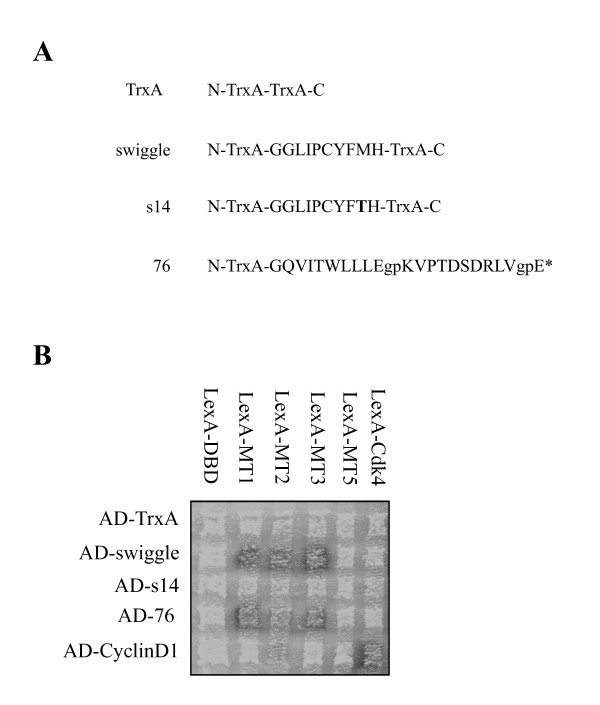
**Isolation and validation of PAs interacting with MT1-MMP ICD**. **(A) **Schematic representation of the TrxA scaffold and the PAs swiggle, 76, and s14. The sequences of the peptide inserted in TrxA (swiggle, s14) or fused to the N-terminal region of TrxA (76) are detailed. The point mutation in the sequence of s14 is depicted in bold and the amino acids in lower cases in PA 76 represent linkers between the multiple peptides. * denotes the stop codon. **(B) **Swiggle interacts with the MT1-MMP ICD in a yeast-two hybrid interaction assay. EGY48 cells expressing LexA-DBD, LexA-MT1, LexA-MT2, LexA-MT3, LexA-MT5 or LexA-Cdk4 were mated with EGY42 cells expressing AD-TrxA, AD-swiggle, AD-s14, AD-76 or AD-CyclinD1 and plated onto selective media.

In order to identify PAs that interact with the MT1-MMP ICD, a yeast two-hybrid screen was performed as previously described [1]. The 21 amino acid ICD of MT1-MMP was fused to the LexA DNA-binding domain (DBD) to generate the bait (LexA-MT1). As prey constructs, we used a library of 10^6 ^unique 10-amino acid residue peptides that were inserted into the active loop of the *E. coli *thioredoxin (TrxA) scaffold protein. The prey constructs were transformed into the EGY42 yeast strain, which was then mated with EGY48 cells expressing LexA-MT1. Replica plating of the resulting diploids to selective media gave rise to 78 colonies where the *LEU2 *reporter gene was activated. From these, only two plasmids were isolated that, when re-transformed into fresh EGY42 cells, still led to the activation of the *LEU2 *and *LacZ *reporter genes in the presence of LexA-MT1. These two PAs were named swiggle and 76. Sequence analysis revealed that, as expected, swiggle comprises a 10 amino acid peptide within TrxA (Figure 1A). PA 76 was found to comprise a 25 amino acid sequence carboxy-terminal to the active loop of TrxA (Figure 1A). Peptides longer than 10 residues arise when two or more peptide-encoding oligonucleotides ligate to each other during library construction. In this case, the 3' of the fused oligonucleotide encodes an in-frame stop codon. In contrast to swiggle, PA 76 is not constrained (in 2 dimensions) at the carboxy-terminus and can be described as a linear peptide fused to the carboxy-terminus of a truncated TrxA fragment (Figure 1A), although it is possible that residues in this "peptide" form ionic or hydrophobic interactions with expressed regions of the TrxA scaffold, leading to at least some element of three-dimensional constraint. Blast searches of Genbank with each PA insert sequence revealed no similarity with known proteins.

To confirm the interaction of the selected PAs with MT1-MMP ICD, yeast interaction mating experiments were performed [44]. Haploid yeast strains expressing the LexA DNA-binding domain alone (LexA-DBD), LexA DBD fusions to the MT1-MMP ICD (LexA-MT1) or Cdk4 (LexA-Cdk4) were mated with strains expressing B42 activation domain (AD) fusions to TrxA (AD-TrxA), swiggle (AD-swiggle), 76 (AD-76) or Cyclin D1 (AD-CyclinD1). We included in this assay a fusion of the AD to a mutant variant of swiggle, called s14, identified by random mutagenesis, which no longer binds to the LexA-MT1 fusion protein. s14 differs from swiggle by a single amino acid: GGLIPCYFMH in swiggle to GGLIPCYFTH in s14 (Figure 1A). Resulting diploids were grown on selective media to identify positive (Figure 1B, grey squares) or negative (Figure 1B, white squares) interactions. As expected, a clear interaction was observed between the LexA-Cdk4 and AD-CyclinD1 pair used as a positive control (Figure 1B). LexA-MT1 did not bind to AD-TrxA, AD-s14 or AD-CyclinD1 but a clear interaction with AD-swiggle or AD-76 (Figure 1B) could be observed confirming that both PAs interact, via the inserted peptide, with the MT1-MMP ICD. In some experiments, a weak interaction between the LexA-DBD and AD-76 could also be observed, suggesting that PA 76 may also recognize sequences or surfaces from the LexA-DBD (data not shown). No interaction was detected between the LexA-DBD and AD-TrxA, AD-swiggle, AD-s14, AD-76 or AD-CyclinD1 (Figure 1B). AD-swiggle was also found to interact with the ICD of MT2-MMP and MT3-MMP (LexA-MT2 and -MT3 in Figure 1B). AD-76 clearly interacted with LexA-MT3 and a weak interaction with LexA-MT2 was observed. AD-swiggle and AD-76 did not interact with the ICD of MT5-MMP (LexA-MT5 in Figure 1B). Taken together, our data clearly demonstrate an interaction between the MT1-MMP ICD and both PAs. The weak interaction between AD-76 and LexA-DBD, coupled with the truncated structure of PA 76 and its instability when expressed in *E. coli *and mammalian cells (data not shown) led us to focus on swiggle in subsequent experiments.

### GFP-swiggle co-immunoprecipitates with MT1-MMP in MCF7 cells

**Figure 2:**
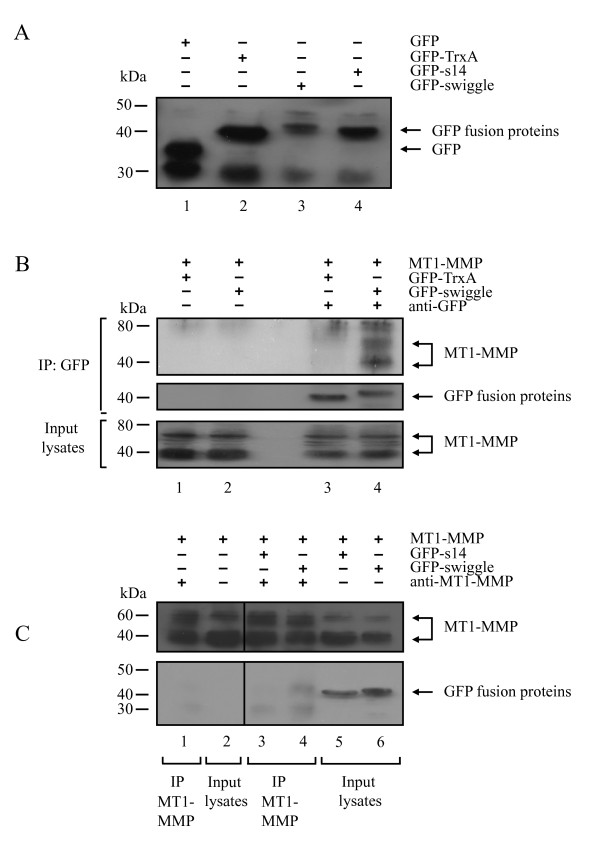
**GFP-swiggle co-immunoprecipitates with MT1-MMP**. **(A) **Western blot analysis of protein extracts prepared from MCF7 expressing GFP (lane 1), GFP-TrxA (lane 2), GFP-s14 (lane 3) or GFP-swiggle (lane 4) using an anti-GFP polyclonal serum. **(B) **Cell lysates prepared from MCF7 cells expressing MT1-MMP and GFP-TrxA (lanes 1 and 3), MT1-MMP and GFP-swiggle (lanes 2 and 4) were immunoprecipitated with an anti-GFP polyclonal antibody (lanes 3 and 4). The presence of MT1-MMP (top panel) and GFP-TRxA and GFP-swiggle (middle panel) in the immunocomplexes was monitored by Western blotting. The expression level of MT1-MMP in the input lysates was analysed by Western blot (bottom panel). **(C) **Cell lysates from MCF7 cells expressing MT1-MMP (lanes 1 and 2), MT1-MMP and GFP-s14 (lanes 3 and 5), MT1-MMP and GFP-swiggle (lanes 4 and 6) were immunoprecipitated with an anti-MT1-MMP pAb (lanes 1, 3 and 4). MT1-MMP, GFP-s14 or GFP-swiggle were detected in the input lysates (lanes 2, 5 and 6) and immunoprecipitated materials (lanes 1, 3 and 4) using an anti-MT1-MMP pAb or an anti-GFP polyclonal serum, respectively.

These interactions between swiggle and the MT1-MMP ICD led us to test whether the two proteins could also interact when co-expressed in mammalian cells. TrxA-based PAs have previously been reported to be occasionally difficult to express in mammalian cells [45]. This was also the case for swiggle with poor expression observed in all the cell lines tested (data not shown). Increased swiggle stability and improved detection by western blot were achieved by fusing GFP to the amino terminus of the PA (Figure 2A). Addition of the tag also allowed detection of the PA by fluorescence microscopy. GFP was also added to the amino terminus of TrxA and s14 to generate GFP-TrxA and GFP-s14, respectively. We used the human MCF7 breast carcinoma cells, which do not naturally express MT1-MMP [46]. mRNA expression for MT2- and MT3-MMP in these cells is very low (our unpublished data and [26]). MCF7 cells were co-transfected with MT1-MMP, and either GFP-swiggle or GFP-TrxA. Total cell lysates were subjected to immunoprecipitation with an anti-GFP antibody followed by Western blot analysis with an anti-MT1-MMP antibody (Figure 2B). No MT1-MMP was found associated with the immunoprecipitates when the anti-GFP antibody was omitted (Figure 2B, lanes 1 and 2, top panel) eliminating the possibility of non-specific binding of the GFP-tagged constructs or MT1-MMP to the beads. MT1-MMP was clearly found to co-immunoprecipitate with GFP-swiggle (Figure 2B, lane 4, top panel and Figure 2C, lane 4, bottom panel), but not with GFP-TrxA (Figure 2B, lane 3, top panel) or GFP-s14 (Figure 2C, lane 3, bottom panel). Together our data demonstrate that swiggle can recognise and interact with the MT1-MMP ICD within human cells.

### GFP-swiggle increases MT1-MMP-mediated cell migration

**Figure 3:**
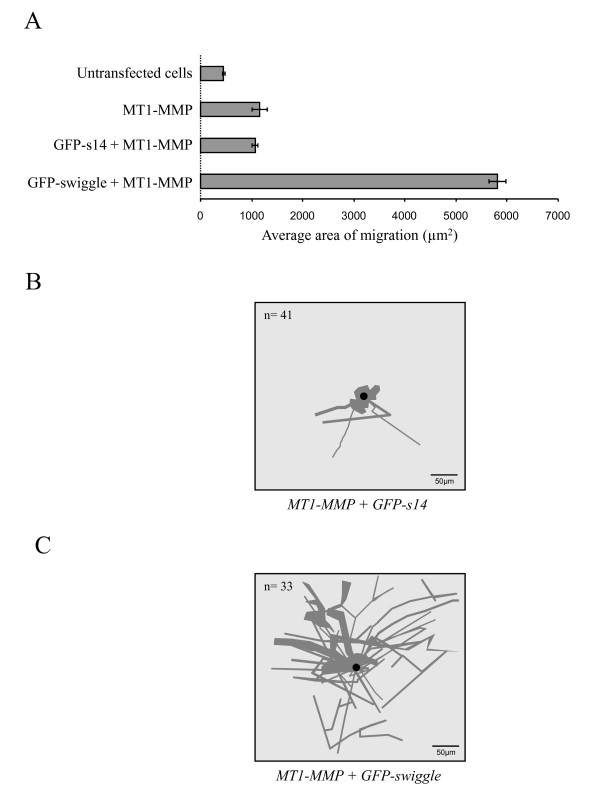
**GFP-swiggle increases MT1-MMP mediated cell migration**. **(A) **Untransfected MCF7 cells or cells expressing MT1-MMP alone or together with GFP-s14, or GFP-swiggle were incubated on colloidal gold-coated coverslips for 24 hours at 37°C. Cells were then fixed and immunostained with an MT1-MMP antibody. The area of migration of at least 50 cells was measured and averaged. Representative phagokinetic tracks generated by MCF7 cells expressing MT1-MMP and **(B) **GFP-s14 or **(C) **GFP-swiggle.

The interaction between the intracellular domain of MT1-MMP and swiggle in human cells led us to ask whether we could detect functional effects of the PA on an ICD-mediated cellular function of the protease. The MT1-MMP ICD has previously been reported to play an important role in promoting cell migration [42,39]. In order to test whether swiggle could alter ICD-dependent MT1-MMP-mediated cell migration, we performed a cell migration assay [18,31,47,33]. Untransfected MCF7 cells as well as cells expressing MT1-MMP alone, MT1-MMP and either GFP-s14 or GFP-swiggle were plated on colloidal gold-coated coverslips. After 24 hours incubation at 37°C, the areas of the phagokinetic tracks generated by cell migration were measured. As expected, very little migration was observed for untransfected MCF7 cells (Figure 3A). Expression of MT1-MMP alone resulted in increased (3 fold in our case) cell migration compared to untransfected cells (Figure 3A) as previously observed [39,42,48]. To our surprise, expression of MT1-MMP and GFP-swiggle (Figures 3A and 3C) resulted in a considerable increase (5.5 fold) in cell migration compared to cells expressing MT1-MMP alone (Figure 3C). Expression of GFP-s14 did not have any effect on MT1-MMP-mediated cell migration (Figures 3A and 3C). All told, cells co-expressing MT1-MMP and swiggle showed a 15-fold enhancement in migration compared to untransfected MCF7 cells. Together, these observations indicate that expression of GFP-swiggle can affect a cellular function of the intracellular domain of MT1-MMP.

### GFP-swiggle increases expression of MT1-MMP at the cell surface

**Figure 4:**
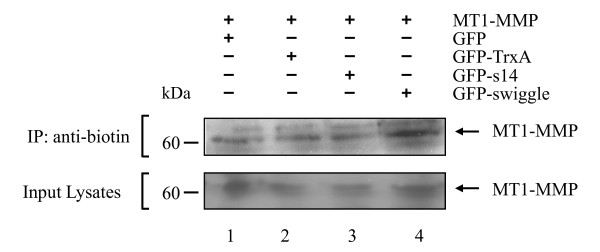
**Expression of GFP-swiggle increases cell surface localisation of MT1-MMP**. Cell surface proteins of MCF7 cells transfected with MT1-MMP, and GFP (lane 1), GFP-TrxA (lane 2), GFP-s14 (lane 3), or GFP-swiggle (lane 4) were biotinylated and immunoprecipitated using an anti-biotin antibody. The presence of MT1-MMP in the immunoprecipitate (top panel) and in the input lysates (bottom panel) was detected using an anti-MT1-MMP antibody.

Another observed role for the ICD is the internalisation of MT1-MMP from the plasma membrane [31,32,49]. The amplification of MT1-MMP-mediated cell migration observed after expression of GFP-swiggle could therefore be the result of an increased expression of the protease at the cell surface. To test this hypothesis, total exposed cell surface proteins of MCF7 cells over-expressing MT1-MMP, and either GFP, GFP-TrxA, GFP-s14 or GFP-swiggle were biotinylated and immunoprecipitated using an anti-biotin antibody. The presence of MT1-MMP was then detected in the complexes by immunoblotting. Inspection of total cell lysates indicated that the absolute levels of MT1-MMP were unaffected by co-expression of any of the GFP fusions. Of this cellular pool, a similar amount of MT1-MMP was detected at the cell surface of MCF7 cells co-expressing GFP (Figure 4, lane 1), GFP-TrxA (Figure 4, lane 2) or GFP-s14 (Figure 4, lane 3). In contrast, the cell surface expression of the protease was clearly increased when GFP-swiggle was co-expressed with MT1-MMP (Figure 4, lane 4).

**Figure 5:**
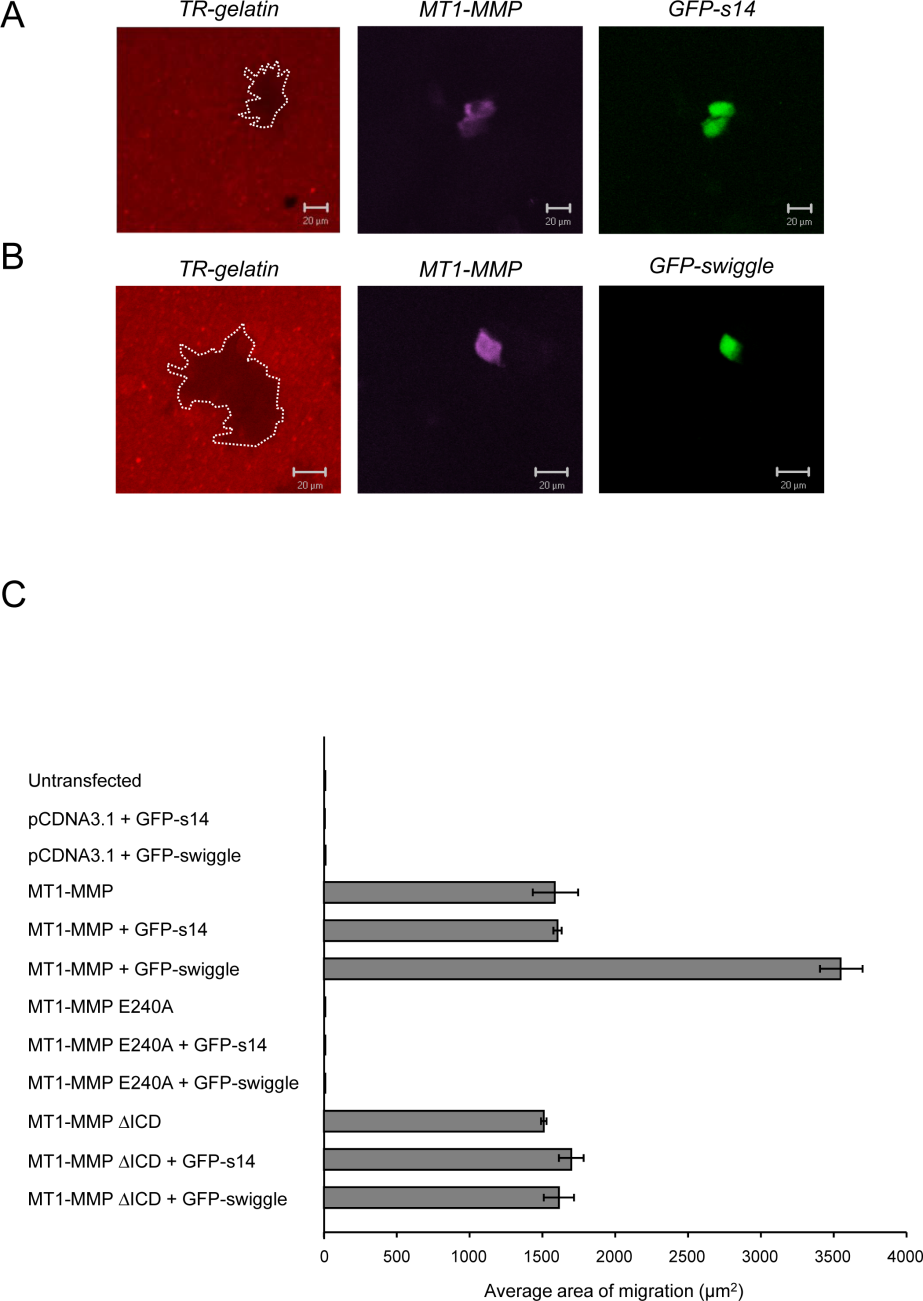
**GFP-swiggle increases MT1-MMP-mediated Texas Red-gelatin degradation**. Example of Texas-Red gelatin degradation (white dotted line) by MCF7 cells expressing MT1-MMP and **(A) **GFP-s14 or **(B) **GFP-swiggle after 16 hours incubation at 37°C. GFP-s14 or GFP-swiggle (green) and MT1-MMP (purple) expression were detected by immunofluorescence. Bar = 20 μm **(C) **Quantification of the area of migration of at least 50 untransfected MCF7 cells or 50 cells transfected with GFP-s14, GFP-swiggle, MT1-MMP, MT1-MMP + GFP-s14, MT1-MMP + GFP-swiggle, MT1-MMP E240A, MT1-MMP E240A + GFP-s14, MT1-MMP E240A + GFP-swiggle, MT1-MMP ΔICD, MT1-MMP ΔICD + GFP-s14 or MT1-MMP ΔICD + GFP-swiggle. Data represent average area of migration ± s.e.m..

The activity of cell-surface localised MT1-MMP can be assessed using a Texas Red-labelled gelatin (TR-gelatin) degradation assay [50]. MCF7 cells transfected with expression constructs for either MT1-MMP, a catalytically inactive MT1-MMP E240A mutant or an ICD-deleted MT1-MMP (MT1-MMP ΔICD) together with either GFP-swiggle or GFP-s14 were plated onto TR-gelatin coated slides and incubated at 37°C. As expected, untransfected MCF7 cells or cells expressing the catalytically inactive MT1-MMP E240A mutant were unable to degrade the TR-gelatin and this result was unaffected by co-expression of either GFP-s14 or GFP-swiggle (Figure 5C). Expression of active MT1-MMP alone in MCF7 cells resulted in a clear degradation of the TR-gelatin (Figure 5C) as previously observed in CHO-K1 and CHO L761 cells expressing the protease [50,51]. A similar level of TR-gelatin degradation was also observed in cells co-expressing MT1-MMP and GFP-s14 (Figures 5A and 5C). In contrast, expression of MT1-MMP together with GFP-swiggle resulted in a 2.5 fold increase in the average area of TR-gelatin degradation per cell (Figures 5B and 5C) compared to MCF7 cells expressing MT1-MMP alone or together with GFP-s-14 (Figure 5C). Increased TR-gelatin degradation was not observed in MCF7 cells expressing an active MT1-MMP with a deleted ICD (MT1-MMP ΔICD) [52] or in cells co-expressing MT1-MMP ΔICD and GFP-swiggle or GFP-s14 (Figure 5C). Together, these data show that GFP-swiggle is able to increase the levels of MT1-MMP activity at the cell surface through interaction with the MT1-MMP ICD. Consistent with the increased level of MT1-MMP protein at the surface of cells expressing GFP-swiggle, these data also confirm that the increase of the protease at the cell surface is mediated by the interaction between GFP-swiggle and the ICD of MT1-MMP.

### Swiggle inhibits MT1-MMP internalization

**Figure 6:**
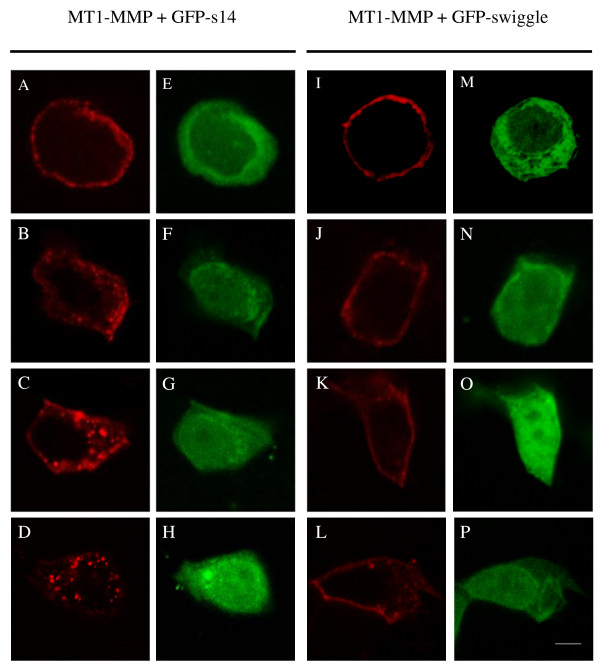
**GFP-swiggle inhibits internalization of MT1-MMP**. MCF7 cells, transiently co-transfected with MT1-MMP and either GFP-s14 **(A-H) **or GFP-swiggle **(I-P) **were incubated with an anti-MT1-MMP antibody for 2 hours at 4°C. Cells were then washed and fixed immediately with paraformaldehyde (A, E, I and M) or warmed to 37°C for 10 (B, F, J and N), 30 (C, G, K and O) or 50 (D, H, L and P) minutes before fixation. MT1-MMP is shown in panels A to D and I to L. GFP-s14 is shown in panels E to H and GFP-swiggle in panels M to P. Bar, 5 μm.

**Figure 7:**
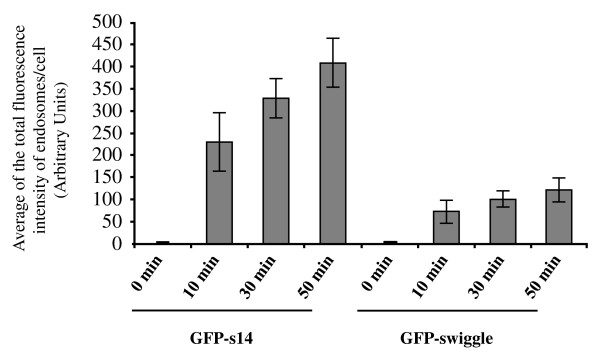
**Quantification of MT1-MMP endocytosis in MCF7 cells transfected with MT1-MMP and GFP-s14 or GFP-swiggle**. MCF7 cells transiently co-transfected with MT1-MMP and either GFP-s14 or GFP-swiggle were incubated with an anti-MT1-MMP antibody for 2 hours at 4°C. Cells were fixed immediately (0 min) or after 10, 30 or 50 minutes at 37°C. Data represent the average of the total fluorescence intensity of endosomes per cell ± s.e.m..

The simplest explanation for the swiggle- and ICD-mediated increased expression of MT1-MMP at the cell surface is that GFP-swiggle expression results in a perturbation of the internalization of the protease [49,31]. We tested whether expression of GFP-swiggle could interfere with the internalization of the protease by performing an antibody internalization assay using the N175 anti-MT1-MMP pAb. This antibody is directed against the whole extracellular domain of the protease and recognises full length as well as the 45 kDa inactive form of the protease [49,50]. MCF7 cells expressing MT1-MMP, and either GFP-s14 (Figure 6, panels A to H) or GFP-swiggle (Figure 6, panels I to P) were incubated with an anti-MT1-MMP antibody on ice, and then warmed to 37°C to restore endocytosis. In cells expressing MT1-MMP alone (data not shown) or MT1-MMP and GFP-s14 (Figure 6, panels A to H), the antibody-bound MT1-MMP originally present at the cell surface (time zero; Figure 6, panel A) was quickly internalized at 37°C. Accumulation of vesicles containing antibody-bound MT1-MMP was clearly observed in the cytoplasm of the cells expressing GFP-s14 (Figure 6, panels B, C and D), demonstrating that the MT1-MMP antibody complex was internalized in these cells. In contrast, in MCF7 cells expressing MT1-MMP and GFP-swiggle (Figure 6, panels I to P), we observed a marked reduction of vesicles containing antibody-bound MT1-MMP, with most of the MT1-MMP antibody complex present at the cell surface when the cells were incubated at 37°C (Figure 6, panels J, K and L). A low level of internalization of antibody bound MT1-MMP complexes could however be detected by 50 minutes (Figure 6, panel L) in cells expressing GFP-swiggle. Quantification of MT1-MMP endocytosis MCF7 cells transfected with MT1-MMP and GFP-s14 or MT1-MMP and GFP-swiggle after 0, 10, 30 or 50 minutes at 37°C is presented in Figure 7.

### Swiggle interacts with the MT1-MMP LLY^573 ^internalization motif

The internalisation of MT1-MMP has been shown to be able to occur via clathrin- or caveolin-mediated pathways [49,53,31,32]. Because MCF7 cells do not express detectable levels of caveolin-1 [54-58], we asked whether GFP-swiggle was able to interfere with the clathrin-mediated internalisation of the protease.

**Figure 8:**
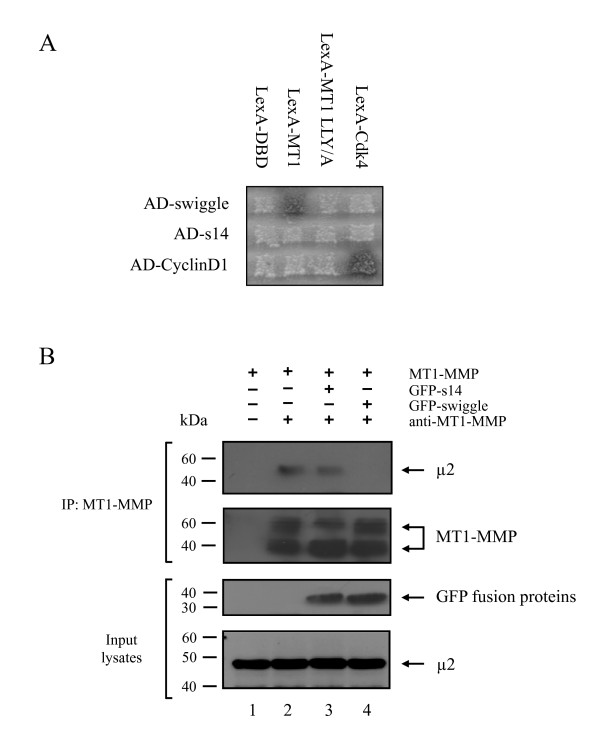
**Swiggle interacts with the LLY^573 ^motif of the MT1-MMP ICD**. **(A) **EGY48 cells expressing LexA-DBD, LexA-MT1, the LexA-MT1 LLY/A mutant or LexA-Cdk4 were mated with EGY42 cells expressing AD-swiggle, AD-s14 or AD-CyclinD1 and plated onto selective media. **(B) **Cell lysates prepared from MCF7 cells expressing MT1-MMP alone (lanes 1 and 2) or together with GFP-s14 (lane 3) or GFP-swiggle (lane 4) were subjected to immunoprecipitation in the presence (lanes 2, 3 and 4) or the absence (lane 1) of an anti-MT1-MMP pAb The presence of μ2 and MT1-MMP in the immunocomplexes was monitored by Western blotting. The expression level of GFP-s14, GFP-swiggle or μ2 in the input lysates was analysed by Western blot.

Clathrin-mediated endocytosis of MT1-MMP has previously been shown to involve the μ2 subunit of the adaptor protein AP-2, which interacts with the LLY^573 ^motif found in the MT1-MMP ICD [31]. Therefore, the inhibition of MT1-MMP internalization observed following expression of GFP-swiggle could potentially result from a disruption of the interaction between MT1-MMP and μ2. The first test of this hypothesis was to ask whether the LLY^573 ^motif required for μ2 binding is also required for binding by swiggle. We therefore generated a LexA-MT1 LLY/A triple mutant, where the LLY^573 ^motif in the MT1-MMP ICD was replaced by AAA^573^, and tested its interaction with AD-swiggle in the yeast two-hybrid interaction assay. As before, we were able to detect a strong interaction in yeast expressing LexA-MT1 and AD-swiggle (Figure 8A; compare to Figure 1B). In contrast, no interaction was found between LexA-MT1 LLY/A and AD-swiggle (Figure 8A) suggesting that the LLY^573 ^motif of MT1-MMP plays a crucial role in the interaction between swiggle and the MT1-MMP ICD.

If swiggle and μ2 both bind to the LLY^573 ^motif of MT1-MMP, then expression of swiggle should competitively inhibit the interaction between μ2 and MT1-MMP in cells. To test this idea, MCF7 cells were transiently co-transfected with MT1-MMP alone or with MT1-MMP together with GFP-s14 or GFP-swiggle. Total cell lysates were subjected to immunoprecipitation with the anti-MT1-MMP antibody and immune complexes were probed with anti-μ2, -MT1-MMP and -GFP antibodies. As shown in Figure 8B, we observed a clear co-immunoprecipitation of MT1-MMP and μ2 when the protease is expressed alone (Figure 8B, lane 2), or in the presence of GFP-s14 (Figure 8B, lane 3). In contrast, we were unable to detect μ2 in complexes immunoprecipitated from MCF7 cells expressing MT1-MMP and GFP-swiggle (Figure 8B, lane 4), thus demonstrating that expression of the PA inhibits the formation of a complex between the protease and the μ2 subunit. Taken together, our data suggest that the inhibition of MT1-MMP endocytosis observed following expression of swiggle probably results from the interaction of the PA with the LLY^573 ^motif of the MT1-MMP ICD, thus disrupting the interaction of the MT1-MMP ICD with the μ2 subunit. Our results thus suggest a potential mechanism for the amplification of the effects of MT1-MMP on cell migration by GFP-swiggle. They also demonstrate that swiggle binding to the ICD can inhibit endocytosis of MT1-MMP without affecting communication between the ICD and the machinery required for cell migration.

## Discussion

In this study, we have described the isolation and characterisation of a PA, called swiggle, which binds to the ICD of the membrane-bound matrix metalloproteinase, MT1-MMP. Our results have several implications for PA technology. Firstly, we have demonstrated that PAs can be successfully obtained against small intracellular domains of transmembrane proteins. Although short peptides have previously been used in yeast two hybrid interaction assays, for example to map interaction domains, the 21 amino acid MT1-MMP ICD bait is approximately three times smaller than the previous smallest published bait used in a screen, a 59 amino acid C-terminal portion of the HPV-16 E7 protein [6]. Our bait is also more than six times smaller than the only other published ICD used in a PA screen, the 133 amino acid ICD of the EGF receptor [7]. Given the dearth of tools to directly study the biology of the MT1-MMP ICD, which could be attributed to its small size, this study raises the possibility that PA technology could be used to generate tools to study the biology of other small intracellular domains, such as integrins, that were previously difficult to study.

PAs have traditionally been found to produce inhibitory phenotypes by blocking certain functions of their target proteins [10,3,11,5,8,13,9]. For example, PAs that interacted with the human papilloma virus (HPV) E6 oncoprotein could block E6-mediated degradation of p53, resulting in increased p53 protein levels, growth inhibition and apoptosis in HPV-positive HPV16 cells [5]. In contrast, we found that expression of GFP-swiggle resulted in an increase in both MT1-MMP-mediated TR-gelatin degradation and cell migration. Furthermore, GFP-swiggle appeared to affect the amount of MT1-MMP at the cell surface. This increased gain of function migratory phenotype caused by GFP-swiggle is unique, although it is consistent with the original prediction for the mechanism of PA function [1] in that it appears to result from a loss of function, namely reduction of the rate of endocytosis of MT1-MMP. Although a previous study has described a PA that stimulates its target protein, calcineurin, this PA was identified from an anti-proliferative phenotypic screen, rather than a yeast-two hybrid screen with a defined bait [59]. The phenotype of this PA is also the opposite of swiggle, with a gain of function of its target resulting in a loss of function phenotype. Together, these studies thus demonstrate the broad applicability of PA technology to the dissection of cell biology.

The increase in MT1-MMP mediated cell migration and TR-gelatin degradation mediated by GFP-swiggle most probably results from the inhibition of endocytosis leading to increased levels of the protease at the cell surface. However, ICD mutants used in other studies that resulted in increased levels of MT1-MMP protein at the cell surface did not cause any effect on cell migration/invasion [30,31,33]. For example, over-expression of wild type MT1-MMP leads to increased cell migration and invasion, but truncations of the ICD including the LLY^573 ^residues, or removal of the whole of the ICD prevented these effects [30,31,33]. This appears paradoxical as truncations that decrease the rate of MT1-MMP internalization should result in more MT1-MMP being present at the cell surface, and hence more proteolytic/migratory activity. One possible explanation for this observation is that the LLY^573 ^motif of the MT1-MMP ICD is required both for clathrin-mediated internalisation and MT1-MMP-mediated cell migration. However, no tools previously existed to test this idea. Our results now show that the expression of GFP-swiggle reduces the rate of internalization of MT1-MMP and results in an increased amount of MT1-MMP at the cell surface, mimicking this aspect of LLY^573 ^mutation. But unlike the ICD LLY^573 ^mutation, GFP-swiggle also allows cell migration to proceed.

A potential caveat of our experiments as well as those of [30,31,33], is that the majority of the assays that were used were only sensitive to over-expressed levels of MT1-MMP. While this may reflect the pathological situation in cancer cells, further work is needed to assess whether GFP-swiggle can perturb other functions of MT1-MMP in normal cells through the development of assays that are sensitive to endogenous levels of MT1-MMP.

## Conclusions

In summary, we have identified and characterized a PA that, when expressed in mammalian cells, can inhibit the clathrin-mediated internalisation of MT1-MMP by interacting with the normal function of the ICD of the protease. In addition to laying the ground-work for the study of the mechanism of endocytic recycling of transmembrane MMPs, this PA should be a useful tool in further studies of MT1-MMP in the existing wide range of biological and disease models.

## Methods

All chemicals were AnalaR grade and were purchased from Sigma Aldrich Chemical Co. (Poole, UK) unless indicated otherwise.

### DNA constructions

Oligonucleotides coding for a PGGG linker followed by MT1-MMP, MT2-MMP, MT3-MMP, or MT5-MMP intracellular domain (ICD) were annealed and cloned downstream of the LexA DNA binding domain (DBD) in pEG202 (Origene, Rockville, USA) to generate LexA-MT1, -MT2, -MT3 and -MT5. The MT1-MMP ICD LLY/A mutant in pEG202 was created by site-directed mutagenesis (Stratagene, La Jolla, CA, USA). Swiggle, and s14, all in pJG4-5 (Origene), were amplified by PCR and subcloned into pIRES2eGFP (BD Clontech, UK). Swiggle and s14 were excised from the above constructs and subcloned into BretGFP-C1 (PerkinElmer, Boston, MA, USA). TrxA, excised from pJM-1 (gift from R. Brent, Berkeley, USA) was subcloned into BretGFP-C2. N-terminally His_6_-tagged swiggle was generated by cloning swiggle into pET32a (Merck Biosciences Ltd., Nottingham, UK).

### Yeast two-hybrid screen

The peptide aptamer (PA) library containing a B42 AD fusion to 1 × 10^6 ^different PAs was constructed as described in [45] and transformed into the reporter *S. cerevisiae *strain EGY48 (*MATα leu2::LexA 6op-LEU2 his3 trp1 ura3*) [60]. These cells were mated with EGY42 cells (*MATa leu2 his3 trp1 ura3*) [10]) carrying LexA-MT1. The two-hybrid mating screen was performed essentially as described by [60]. Interactors, selected on Ura^-^His^-^Trp^-^Leu^-^/X-Gal, Gal, Raff plates after four days at 30°C, were picked and the plasmids rescued into *E. coli *KC8 cells. The plasmids were transformed back into EGY48 to confirm interaction with LexA-MT1 in EGY42 using an interaction-mating matrix. LexA-Cdk4 and AR cDNAs were kindly provided by P. Hinds and M. Lu (Harvard Medical School, USA). AD-CyclinD1 cDNA was from R. Brent (Molecular Sciences Institute, Berkeley CA, USA).

### Isolation of mutants of swiggle by PCR mutagenesis of the swiggle peptide insert

PCR mutagenesis of the swiggle peptide insert was performed as previously described [61] except for the following alterations. Mutated swiggle sequences were ligated into *Rsr*II-cut pJM-1 vector and were transformed into XL-10 gold *E. coli *cells (Invitrogen Ltd., Paisley, UK). Plasmid DNA was isolated using Qiagen midi-prep DNA extraction kit (Qiagen, Crawley, UK), and transformed (1 μg) into EGY48 containing LexA-MT1 and pJK103 [61]. Positive (blue) and negative (white) interactors were selected on Ura^-^His^-^Trp^-^Leu^-^/X-Gal, Gal, Raff plates for four days, picked and the plasmids rescued into *E. coli *KC8 cells. Plasmids were transformed back into EGY48 to confirm interaction with LexA-MT1 in EGY42 using an interaction-mating matrix.

### Cell culture and transfections

All cell culture reagents were purchased from Invitrogen Ltd. unless indicated. MCF7 cells, purchased from ECACC (Salisbury, UK), were maintained in DMEM, supplemented with 10% (vol/vol) fetal calf serum (FCS, Hyclone Laboratories, UT, USA) and 2 mM L-glutamine at 37°C in 5% CO2 atmosphere. Transfections were performed using FuGENE-6 reagent (Roche Diagnostics, Lewes, UK) according to manufacturer's instructions.

### Immunoprecipitation and western blotting

MCF7 cells were transfected for 48 hours with either 0.4 μg GFP-swiggle and 0.6 μg MT1-MMP constructs, or 0.4 μg GFP-TrxA and 0.6 μg MT1-MMP in 6-well plates. After 2 washes with ice-cold PBS, cells were lysed for 15 minutes on ice under rocking conditions in 200 μl of lysis buffer (50 mM Tris-HCl, pH 7.4, 100 mM NaCl, 2 mM EDTA, 1% Triton X-100, 60 mM octyl-D-glucoside) supplemented with complete protease inhibitor cocktail (Roche Molecular Biochemical, Hertfordshire, UK), sonicated (80 Volts for 10 seconds, Sonics and Material Inc., Suffolk, UK) and centrifugated (10 minutes at 15000 g at 4°C). An aliquot (10%) of the protein extract was kept and analysed by Western blot (input lysate). The remaining protein extract was incubated for 2 hours at 4°C with an anti-GFP rabbit polyclonal serum (dilution 1/2200). Protein G sepharose beads (20 μl of slurry) were then added and the mixture rotated for a further 2 hours at 4°C. Beads were washed four times with 1 × PBS, once with PBS diluted 5 times in distilled water and finally were resuspended in Laemmli sample buffer (LSB). Immunoprecipitates and input lysates were boiled for 5 minutes, resolved on 12% SDS-polyacrylamide gels, and electrotransferred onto PVDF membrane (Millipore, Watford, UK). Western blots were carried out as previously described [45]. Rabbit anti-GFP serum (Abcam, Cambridge, UK) was used at a 1:2000 dilution. The anti-MT1-MMP sheep N175 pAb, directed against the entire extracellular of the protease [49,50], was used at 10 μg/ml.

### Cell surface biotinylation of proteins

Cell surface biotinylation was carried out as previously described [49], except for the following alterations. MCF7 cells (1 × 10^5^) were transfected with 0.4 μg GFP-swiggle and 0.6 μg MT1-MMP, 0.4 μg GFP-TrxA and 0.6 μg MT1-MMP, 0.4 μg GFP-s14 and 0.6 μg MT1-MMP, or 0.4 μg GFP and 0.6 μg MT1-MMP in 6-well plates. PBS buffer was substituted for Soerensen buffer (SBS). Cells were incubated for 30 minutes in ice-cold PBS containing 0.5 mg/ml of NHS-SS-Biotin (Pierce Biochemical, Rockford, USA) and lyzed for 15 minutes at 4°C in 200 μl ice-cold RIPA buffer containing protease inhibitor III cocktail (Calbiochem Biochemical, West Drayton, UK). The lysate was cleared by centrifugation (16000 g for 5 minutes at 4°C) and incubation with protein A/G plus-agarose beads (25 μl; Santa Cruz Biotechnology, Santa Cruz, USA) for 1 hour at 4°C. An aliquot of the whole cell lysate (20 μl; input lysate) was kept for western blot analysis. Anti-biotin mAb (12 μg; Jackson Immunoresearch Ltd., Soham, UK) and Protein A/G Plus agarose beads (30 μl) were then added to the extract and incubated for 1 hour at 4°C under constant rotation. Beads were washed five times with ice-cold RIPA buffer, resuspended in LSB and processed for western blotting as previously described.

### Immunofluorescence microscopy

Cells seeded on glass coverslips were washed in PBS, fixed at room temperature (RT) in 4% (wt/vol) paraformaldehyde (PFA, BDH, Poole, UK) for 5 minutes and washed twice with PBS. For permeabilized cells, coverslips were incubated in 0.2% Triton-X100 in PBS for 5 minutes at RT, and washed three times in 2 × PBS. Cells were blocked for 30 minutes at RT with PBS containing 10% FCS and 10 μg/ml BSA, and then incubated with the anti-MT1-MMP sheep pAb (10 μg/ml) for 16 hours at 4°C in PBS containing 10 μg/ml BSA. After 3 washes in PBS, cells were incubated for 1 hour at RT with fluorescently-conjugated secondary antibody (Jackson Immunoresearch Ltd.) in PBS and according to the manufacturer's instructions. Coverslips were washed repeatedly in PBS, and mounted onto glass slides using Vectashield containing diamidino-2-phenylindole (DAPI, Vector Laboratories, Burlingame, USA). Images of fluorescently labelled cells were collected using a Zeiss LSM510 Metaconfocal microscope in a single focal plane (Carl Zeiss Ltd., Welwyn Garden City, UK).

### Texas Red-labelled Gelatin Degradation Assay

Texas Red labelled-gelatin was coated, as previously described by [50] on the glass surface of 8 well Labtek culture slides (Becton Dickinson Labware, USA) and incubated for 2 hours at 37°C in DMEM containing 10% FCS. Untransfected MCF7 cells (5 × 10^4 ^per well) were then seeded on the fluorescent gelatin and incubated for 16 hours at 37°C. Cell transfections were carried out using FuGENE-6 (0.75 μl) and 250 ng of DNA (ratio used). Cells were then washed in warmed PBS, fixed with 4% (w/v) PFA in PBS and then processed immunofluorescence microscopy using the anti-MT1-MMP sheep pAb as described above. A Cy5 conjugated donkey anti-sheep secondary antibody (1:200 dilution) was used (Jackson Immunoresearch).

For each condition, pictures of at least 50 cells in a single focal plane were taken with a Zeiss 510 Meta confocal microscope. The area of TR-gelatin degradation was then measured with Zeiss AIM software (version 3.2).

### Phagokinetic track colloidal gold cell migration assay

The phagokinetic track colloidal gold cell migration assay was performed as described previously [18]. Colloidal gold-coated coverslips were placed in a 12-well plate, and transfected MCF7 cells were seeded at 2 × 10^3 ^cells per well. After 24 hours incubation at 37°C, cells were fixed as previously described and phagokinetic tracks were visualized using bright field illumination with a Zeiss 510 Meta confocal microscope. The area of migration for at least 50 transfected cells was measured with Zeiss AIM software and averaged.

### MT1-MMP antibody internalization assay

MCF7 cells, co-transfected with GFP-swiggle (0.4 μg) and MT1-MMP (0.6 μg), or GFP-s14 (0.4 μg) and MT1-MMP (0.6 μg), were seeded on glass coverslips in 6-well plates. After 48 hours transfection, cells were washed twice with ice-cold PBS and incubated with the affinity purified N175 anti-MT1-MMP sheep pAb (5 μg/ml) at 4°C for 2 hours. Coverslips were then washed twice with ice-cold PBS to remove unbound antibody and fixed immediately (zero minute timepoint) in 4% PFA for 10 minutes or placed at 37°C for 10, 30 and 50 minutes in prewarmed media before fixation. Cells were permeabilized and processed for immuno-fluorescence microscopy as previously described. Endocytosis was quantified by measuring the fluorescence intensity of all endocytic vesicles per cell using Metamorph imaging software version 6.1 (Molecular Devices Ltd., Wokingham, UK) as previously described by [62]. At least 5 cells were used for time 0 and 15 cells for the other time points.

## Abbreviations

PA: peptide aptamer; ECM: Extra-cellular matrix; MT1-MMP: membrane-type I matrix metalloproteinase; ICD: intracellular domain; DBD: DNA-binding domain; AD: activation domain.

## Authors' contributions

RDW carried out the molecular genetic studies, participated in the sequence alignment and drafted the manuscript. PKF conceived the study, participated in its design and coordination and helped to draft the manuscript. CR participated in the design and coordination of the study and helped to draft the manuscript. All authors read and approved the final manuscript.
